# Global, regional, and national burden of non-communicable diseases attributable to occupational asbestos exposure 1990–2019 and prediction to 2035: worsening or improving?

**DOI:** 10.1186/s12889-024-18099-4

**Published:** 2024-03-18

**Authors:** Xinlu Miao, Teng Yao, Chenxian Dong, Zuhai Chen, Wanting Wei, Zhengyang Shi, Tongtong Xu, Jianjiang Shao, Qiang Niu, Dongsheng Rui, Yunhua Hu, Yizhong Yan

**Affiliations:** 1 Department of Preventive Medicine, School of Medicine, Shihezi University, No. 59, North 2nd Rd, Hong-Shan District, Shihezi, Xinjiang 832003 China; 2 Key Laboratory for Prevention and Control of Crucial Emerging Infectious Diseases and Public Health Security of The Xinjiang Production and Construction Corps, Shihezi, Xinjiang China; 3 Key Laboratory of Preventive Medicine, Shihezi University, Shihezi, Xinjiang China; 4 Key Laboratory of Xinjiang Endemic and Ethnic Diseases (Ministry of Education), School of Medicine, Shihezi University, Shihezi, Xinjiang China

**Keywords:** Noninfectious diseases, Exposed to asbestos, Epidemiology, Disease burden, Prevention

## Abstract

Understanding the burden associated with occupational asbestos exposure on a global and regional scale is necessary to implement coordinated prevention and control strategies. By the GBD Study 2019, we conducted a comprehensive assessment of the non-communicable diseases burden attributable to occupational asbestos exposure. In 2019, 239,330 deaths and 4,189,000 disability-adjusted life years (DALYs) worldwide due to occupational asbestos exposure occurred. 1990–2019, deaths and DALYs attributed to occupational asbestos exposure increased by 65.65% and 43.66%, respectively. Age-standardized mortality rate (ASMR) and age-standardized DALYs rate (ASDR) decreased, with the most rapid declines in high Socio-Demographic Index (SDI) regions, with average annual percent change (AAPC) of − 1.05(95%CI: −1.2, −0.89) and −1.53(95%CI: −1.71, −1.36), respectively. Lung cancer, mesothelioma and ovarian cancer were the top three contributors to the increase in deaths and DALYs, accounting for more than 96%. AAPCs of ASMR and ASDR were positively associated with SDI. Global deaths from occupational asbestos exposure were predicted to increase and ASMR to decrease by 2035, mostly in males. Due consideration should be given to the susceptibility of the elderly, the lag of asbestos onset, and the regional differences, and constantly improve the prevention and control measures of occupational asbestos exposure and related diseases.

**Supplementary Information:**

The online version contains supplementary material available at 10.1186/s12889-024-18099-4.

## Introduction

Asbestos is defined as a Group I carcinogen by the International Agency for Research on Cancer (IARC). It is one of the most important carcinogens, more than half of occupational cancer deaths are related to asbestos [1, 2]. Because of its good thermal stability, flexibility, wear resistance and corrosion resistance, asbestos has a wide range of applications, such as roof cover, textiles, electrical insulation, cement pipes, friction materials (clutch pads, brake pads), etc [3]. The widespread use of asbestos has increased occupational and environmental exposure to populations. According to the World Health Organization (WHO), about 125 million people worldwide are highly exposed to asbestos at work. More than 255,000 people die each year from asbestos-related diseases [4]. With increasing concern about the health effects of asbestos products, 66 countries have banned the production and use of all types of asbestos [5]. In developing countries where chrysotile is widely used, policies have been adopted to control the use of asbestos in the occupational environment. China has centralized supervision over relevant enterprises and actively promoted the development of asbestos substitutes [6]. Mongolia has issued relevant resolutions to restrict the scope of application of asbestos and only allow the use of asbestos in thermal power plants [7]. Russia, Kazakhstan and other countries have also introduced national plans and carry out epidemiological investigation on asbestos [8].

Exposure to asbestos at any level is not safe. Asbestos fibers are mainly inhaled with air, and have local carcinogenic effects on target organs (lungs, larynx, ovaries) and related serous membranes (pleura, pericardium, peritoneum, vaginal membranes), resulting in lung cancer, larynx cancer, ovarian cancer, pleura, and peritoneal mesothelioma. Asbestos-induced lung cancer accounts for 55–85% of occupational cancers [9]. In addition, the fibrotic effect of asbestos can also develop into pulmonary asbestosis [3]. One-quarter of asbestos-exposed people had CT evidence of asbestosis [10]. Due to their extreme biological persistence, asbestos fibers, which cannot be effectively cleared by macrophages, cause continuous irritation to the lungs and lead to chronic inflammation, so they can remain in the human body for years [11]. Ovarian cancer caused by asbestos exposure has been designated as the first gynecological occupational disease in Germany, and the risk of ovarian cancer was approximately doubled in females with occupational asbestos exposure [12]. Of the many mesothelioma carcinogens (erionite, fluoro-edenite fibrous amphibole and occupational exposure to firefighters and painters), asbestos exposure is the more commonly recognized cause, with 80% of mesotheliomas primarily caused by exposure to asbestos [13]. Due to its long latency period of 30–43.9 years, mesothelioma is typically diagnosed in elderly individuals [14]. This highlights the different health problems and diseases that occupational asbestos exposure can cause in humans.

Understanding the trend of the burden caused by occupational asbestos exposure over time and space is crucial for better constructing occupational asbestos exposure prevention and control regulatory systems and reducing the risk of the asbestos working environment. At the same time, we need to make it clear that asbestos exposure is not only an occupational problem, but also a risk for the general population, and the potential impact of non-occupational asbestos exposure (residents living near asbestos mines due to air pollution, improper disposal of asbestos-containing construction waste, household exposure, etc.) should be taken into account [15]. However, no studies have been conducted to comprehensively and systematically report the burden of disease due to occupational asbestos exposure. Therefore, this study obtained the latest data from the GBD 2019 database to analyze and compare the deaths and disability-adjusted life years (DALYs) of non-communicable diseases (NCDs) (lung cancer, mesothelioma, ovarian cancer, larynx cancer, and asbestosis) due to occupational asbestos exposure, aiming to promote the governments of various countries to timely introduce regulatory policies, particularly arouse the attention and provide the management basis for the countries where asbestos is mined and the countries where asbestos is still used and increase investment in health and safety education activities for special groups, to jointly create a good and safe working environment.

## Materials and methods

### Data collection

By collecting all kinds of published disease data, literature data, clinical research data, etc, GBD carries out comprehensive analysis of various diseases, injuries, and risk factors. GBD 2019 further expands the types of diseases and injuries, covering 369 diseases and 87 risk factors from 204 countries or territories and 21 GBD regions between January 1, 1990, and December 31, 2019 [16, 17].

This study selected information on the burden of NCDs attributable to occupational asbestos exposure, including deaths and DALYs, and corresponding population attribution fraction (PAF) and age-standardized rates (age-standardized mortality rate (ASMR) and age-standardized DALYs rate (ASDR)). Time trends of the burden from five diseases (lung cancer, mesothelioma, ovarian cancer, larynx cancer, and asbestosis) caused by occupational asbestos exposure were analyzed by sex, age, and regional differences were compared. Further, the above countries and territories were classified into five categories based on socio-demographic index (SDI): low SDI, low-middle SDI, middle SDI, high-middle SDI, and high SDI [17].

### Data analysis

We used different health indicators from the GBD 2019 database to analyze the health effects of occupational asbestos exposure, and all indicators contain a 95% uncertainty interval (95% UI).

The population attribution fraction (PAF) represents the proportion of people with a reduced risk of illness or death in a given year if the risk of a certain exposure is reduced or eliminated [18].

$$ PAF=\frac{{P}_{e }\left(RR-1\right)}{{P}_{e}\left(RR-1\right)+1}$$

In the formula, *RR* represents the relative risk from occupational asbestos exposure, and *P*_*e*_ represents deaths from occupational asbestos exposure in the population.

A Jointpoint regression model was constructed using the method of previous study [19], in which a log-linear regression model was used to estimate the average annual percent change (AAPC) and its 95% confidence interval (CI) reflecting the trend of ASMR and ASDR from 1990 to 2019. In addition, we used Pearson correlation analysis to further understand the association between SDI and the burden of NCDs associated with occupational asbestos exposure at the national and regional levels.

We used method from previous study to construct a bayesian age-period cohort (BAPC) model to predict the number of deaths and ASMR of NCDs attributable to occupational asbestos exposure in 2020–2035 [20]. For prediction, the BAPC model has a low error rate and a high coverage rate, and the specific method has been described in the previous studies [21, 22]. In addition, to compare the predictions, we used the 2019 data as a baseline, with a 1% increase in the number of deaths per year as a negative reference and a 1% decrease as a positive reference.

All data in this study were processed by R software version 4.2.1, and a *P* value less than 0.05 indicated that the difference was statistically significant.

## Results

### Global burden of NCDs due to occupational asbestos exposure, 2019

Globally, 239,330 (95%UI: 179,520, 299,210) deaths were caused by occupational asbestos exposure in 2019, among which 40,790 (95%UI: 26,140, 53,160) were females and 198,550 (95%UI: 141,470, 258,120) were males (Table 1). DALYs attributed to occupational asbestos exposure were 4,189,000 (95%UI: 3,127,000, 5,320,000), including 704,000 (95%UI: 467,000, 905,000) for females and 3,485,000 (95%UI: 2,454,000, 4,563,000) for males (Table S1).

**Table 1:** Global deaths attributable to occupational asbestos exposure in 1990 and 2019, and the temporal trend from 1990 to 2019

Cause of deaths	1990			2019			1990–2019	
	Deaths No.×10^3^ (95%UI)	ASMR per 100,000 (95%UI)	Age-standardized PAF,% (95%UI)	Deaths No.×10^3^ (95%UI)	ASMR per 100,000 (95%UI)	Age-standardized PAF,% (95%UI)	AAPC of ASMR (95%CI)	AAPC of Age-standardized PAF (95%CI)
**Overall**	144.48 (107.16, 182.09)	3.97 (2.97, 4.98)	0.36 (0.27, 0.45)	239.33 (179.52, 299.21)	3.05 (2.29, 3.82)	0.42 (0.31, 0.52)	− 0.92 (− 1.12, − 0.73)	0.52 (0.33, 0.72)
**Sex**								
Female	20.62 (14.64, 27.47)	1.01 (0.72, 1.34)	0.10 (0.07, 0.14)	40.79 (26.14, 53.16)	0.93 (0.60, 1.21)	0.15 (0.10, 0.2)	− 0.28 (− 0.46, − 0.09)	1.25 (1.10, 1.39)
Male	123.86 (88.46, 160.07)	8.17 (5.87, 10.49)	0.63 (0.45, 0.81)	198.55 (141.47, 258.12)	5.88 (4.18, 7.59)	0.67 (0.48, 0.87)	− 1.13 (− 1.32, − 0.95)	0.25 (0.13, 0.36)
**Disease type**								
**All**	144.48 (107.16, 182.09)	3.97 (2.97, 4.98)	0.54 (0.41, 0.68)	239.33 (179.52, 299.21)	3.05 (2.29, 3.82)	0.57 (0.42, 0.71)	− 0.92 (− 1.12, − 0.73)	0.13 (− 0.01, 0.28)
**Neoplasms**	143.02 (105.80, 180.41)	3.93 (2.93, 4.94)	2.66 (1.99, 3.35)	235.76 (176.17, 295.88)	3.01 (2.24, 3.77)	2.4 (1.79, 3.02)	− 0.94 (− 1.13, − 0.75)	− 0.36 (− 0.53, − 0.19)
Larynx cancer	2.74 (1.52, 4.10)	0.08 (0.04, 0.11)	3.45 (1.93, 5.11)	3.68 (2.04, 5.53)	0.05 (0.03, 0.07)	3.12 (1.75, 4.64)	− 1.62 (− 1.77, − 1.47)	− 0.34 (− 0.48, − 0.19)
Tracheal, bronchus, and lung cancer	122.21 (86.78, 158.14)	3.37 (2.41, 4.34)	12.34 (8.73, 15.89)	198.7 (140.37, 257.41)	2.54 (1.80, 3.28)	10.09 (7.14, 12.97)	− 0.99 (− 1.20, − 0.78)	− 0.71 (− 0.77, − 0.65)
Ovarian cancer	4.03 (1.86, 6.54)	0.12 (0.05, 0.19)	4.64 (2.14, 7.50)	6.56 (2.95, 10.66)	0.08 (0.04, 0.14)	3.47 (1.61, 5.69)	− 1.12 (− 1.33, − 0.91)	− 1.04 (− 1.20, − 0.88)
Mesothelioma	14.04 (12.48, 15.70)	0.37 (0.33, 0.42)	92.74 (90.96, 94.33)	26.82 (24.31, 28.62)	0.33 (0.30, 0.36)	91.91 (90.02, 93.59)	− 0.37 (− 0.47, − 0.27)	− 0.03 (− 0.04, − 0.02)
**Chronic respiratory diseases**	1.46 (1.18, 1.75)	0.04 (0.03, 0.05)	0.05 (0.04, 0.06)	3.57 (2.58, 4.05)	0.05 (0.03, 0.05)	0.09 (0.06, 0.10)	0.49 (0.22, 0.75)	2.39 (2.17, 2.62)
Asbestosis	1.46 (1.18, 1.75)	0.04 (0.03, 0.05)	6.50 (5.23, 8.03)	3.57 (2.58, 4.05)	0.05 (0.03, 0.05)	16.04 (10.91, 18.10)	0.49 (0.22, 0.75)	3.23 (3.09, 3.36)
**Location**								
**SDI region**								
High SDI	100.2 (75.02, 123.86)	9.21 (6.88, 11.40)	1.36 (1.02, 1.68)	141.21 (107.98, 174.15)	6.77 (5.18, 8.37)	1.51 (1.16, 1.87)	− 1.05 (− 1.20, − 0.89)	0.37 (0.25, 0.48)
High-middle SDI	32.22 (23.49, 41.54)	3.11 (2.27, 3.99)	0.32 (0.24, 0.41)	56.64 (40.97, 73.96)	2.78 (2.01, 3.62)	0.46 (0.33, 0.59)	− 0.39 (− 0.77, 0.00)	1.18 (1.03, 1.33)
Middle SDI	7.72 (5.50, 10.63)	0.90 (0.65, 1.24)	0.08 (0.06, 0.11)	26.9 (18.86, 37.06)	1.23 (0.86, 1.69)	0.17 (0.12, 0.23)	1.08 (0.80, 1.37)	2.51 (2.37, 2.65)
Low-middle SDI	3.06 (2.08, 4.78)	0.61 (0.42, 0.95)	0.04 (0.03, 0.07)	11.54 (8.27, 15.31)	0.95 (0.68, 1.26)	0.10 (0.07, 0.13)	1.57 (1.23, 1.92)	3.16 (2.99, 3.32)
Low SDI	1.20 (0.61, 2.96)	0.58 (0.30, 1.45)	0.03 (0.02, 0.08)	2.93 (1.73, 6.26)	0.68 (0.40, 1.44)	0.06 (0.04, 0.13)	0.49 (0.34, 0.64)	2.12 (1.87, 2.37)
**GBD region**								
Andean Latin America	0.37 (0.20, 0.54)	1.99 (1.07, 2.92)	0.21 (0.11, 0.31)	0.51 (0.32, 0.77)	0.97 (0.60, 1.46)	0.17 (0.11, 0.23)	− 2.42 (− 3.16, − 1.68)	− 0.73 (− 2.04, 0.59)
Australasia	3.37 (2.65, 4.04)	13.86 (10.88, 16.61)	2.14 (1.68, 2.56)	5.1 (4.11, 6.01)	9.53 (7.69, 11.27)	2.41 (1.95, 2.83)	− 1.26 (− 1.47, − 1.05)	0.37 (0.26, 0.47)
Caribbean	0.30 (0.20, 0.41)	1.20 (0.81, 1.66)	0.13 (0.09, 0.18)	0.53 (0.34, 0.77)	1.02 (0.65, 1.49)	0.13 (0.09, 0.19)	− 0.47 (− 1.24, 0.30)	− 0.07 (− 0.48, 0.34)
Central Asia	0.66 (0.42, 0.99)	1.41 (0.91, 2.06)	0.14 (0.09, 0.20)	0.94 (0.64, 1.29)	1.43 (0.98, 1.96)	0.14 (0.10, 0.19)	0.08 (− 0.52, 0.68)	0.20 (− 0.16, 0.56)
Central Europe	2.86 (1.93, 3.97)	1.88 (1.28, 2.60)	0.19 (0.13, 0.26)	7.73 (5.06, 10.99)	3.42 (2.23, 4.86)	0.52 (0.36,0.73)	2.07 (1.74, 2.41)	3.60 (3.07, 4.12)
Central Latin America	0.7 (0.52, 0.91)	0.94 (0.69, 1.23)	0.11 (0.08, 0.14)	2.03 (1.44, 2.74)	0.89 (0.64, 1.21)	0.14 (0.11, 0.19)	− 0.24 (− 0.99, 0.52)	0.88 (0.18, 1.59)
Central Sub-Saharan Africa	0.22 (0.09, 0.72)	1.16 (0.45, 3.76)	0.06 (0.02, 0.20)	0.43 (0.16, 1.32)	1.00 (0.38, 3.07)	0.08 (0.03, 0.25)	− 0.5 (− 0.70, − 0.30)	0.89 (0.75, 1.04)
East Asia	6.49 (4.34, 9.7)	0.92 (0.62, 1.37)	0.08 (0.06, 0.12)	27.16 (17.91, 39.36)	1.47 (0.97, 2.10)	0.23 (0.16, 0.33)	1.58 (1.25, 1.91)	3.58 (3.19, 3.96)
Eastern Europe	5.27 (3.63, 7.26)	1.83 (1.26, 2.51)	0.18 (0.13, 0.25)	6.34 (4.28, 8.79)	1.8 (1.22, 2.49)	0.21 (0.15, 0.29)	0.11 (− 1.16, 1.4)	0.77 (0.15, 1.39)
Eastern Sub-Saharan Africa	0.52 (0.22, 1.64)	0.79 (0.33, 2.49)	0.04 (0.02, 0.13)	1.12 (0.48, 3.52)	0.83 (0.35, 2.59)	0.07 (0.03, 0.22)	0.15 (0.05, 0.24)	2.10 (1.85, 2.35)
High-income Asia Pacific	6.54 (4.58, 8.6)	3.47 (2.45, 4.54)	0.58 (0.41, 0.76)	22.61 (15.55, 29.83)	4.05 (2.80, 5.35)	1.21 (0.84, 1.60)	0.52 (0.08, 0.95)	2.55 (2.21, 2.90)
High-income North America	38.04 (27.91, 47.52)	10.16 (7.43, 12.74)	1.53 (1.12, 1.92)	47.21 (35.47, 59.26)	7.06 (5.30, 8.87)	1.37 (1.03, 1.72)	− 1.20 (− 1.43, − 0.98)	− 0.38 (− 0.51, − 0.26)
North Africa and Middle East	3.51 (2.20, 5.07)	2.21 (1.43, 3.17)	0.19 (0.12, 0.27)	7.18 (4.36, 10.97)	1.90 (1.18, 2.86)	0.25 (0.15, 0.36)	− 0.58 (− 1.18, 0.04)	0.73 (0.23, 1.23)
Oceania	0.03 (0.02, 0.05)	1.30 (0.81, 2.06)	0.10 (0.06, 0.15)	0.11 (0.06, 0.18)	1.86 (1.11, 3.06)	0.15 (0.09,0.23)	1.24 (1.09, 1.39)	1.55 (1.35, 1.74)
South Asia	2.50 (1.62, 4.09)	0.55 (0.36, 0.90)	0.04 (0.02, 0.06)	9.51 (6.72, 13.23)	0.77 (0.55, 1.06)	0.08 (0.06, 0.11)	1.22 (0.45, 1.99)	3.07 (2.82, 3.32)
Southeast Asia	1.64 (1.07, 2.37)	0.72 (0.47, 1.05)	0.06 (0.04, 0.09)	5.20 (3.32, 7.75)	1.00 (0.63, 1.48)	0.12 (0.08, 0.18)	1.21 (0.81, 1.62)	2.41 (1.92, 2.91)
Southern Latin America	1.18 (0.80, 1.59)	2.59 (1.76, 3.50)	0.32 (0.21, 0.43)	2.48 (1.65, 3.40)	2.90 (1.93, 3.98)	0.48 (0.33,0.66)	0.50 (− 0.10, 1.11)	1.48 (1.07, 1.90)
Southern Sub-Saharan Africa	1.16 (0.81, 1.62)	4.59 (3.23, 6.45)	0.40 (0.27, 0.56)	2.26 (1.69, 2.88)	4.50 (3.36, 5.67)	0.37 (0.28, 0.47)	− 0.08 (− 0.85, 0.69)	− 0.27 (− 0.73, 0.18)
Tropical Latin America	1.57 (1.20, 1.99)	2.01 (1.52, 2.54)	0.20 (0.15, 0.25)	4.35 (3.32, 5.45)	1.89 (1.43, 2.37)	0.30 (0.23, 0.38)	− 0.19 (− 0.40, 0.03)	1.43 (1.21, 1.65)
Western Europe	67.24 (51.12, 82.28)	11.09 (8.39, 13.61)	1.63 (1.24, 2.01)	85.99 (67.03, 105.05)	8.68 (6.71, 10.64)	2.05 (1.59, 2.52)	− 0.85 (− 0.95,− 0.75)	0.79 (0.68, 0.90)
Western Sub-Saharan Africa	0.31 (0.17, 0.59)	0.39 (0.22, 0.74)	0.02 (0.01, 0.04)	0.54 (0.32, 1.01)	0.34 (0.20, 0.63)	0.03 (0.02, 0.05)	− 0.42 (− 0.63, − 0.21)	0.76 (0.52, 1.00)

*Note* ASMR, age-standardized mortality rate; PAF, population attributable fraction; AAPC, average annual percentage change; UI, uncertainty interval; CI, confidence interval

**Fig. 1:**
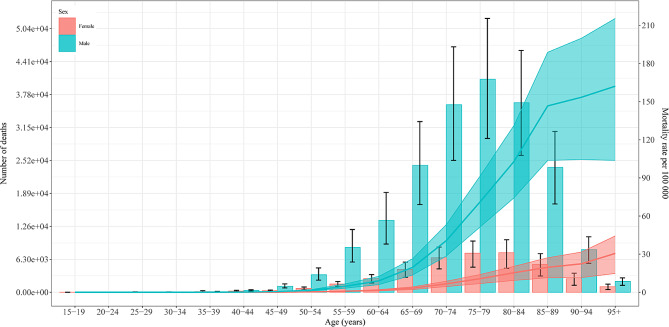
Age-specific numbers (bar plot) and rates (line plot) of deaths attributable to occupational asbestos exposure in 2019 by sex

The age distribution of deaths was unimodal, with a peak of 75–79 years for males and 80–84 years for females. In the 15–39 age range, more females died than males, but the trend has since reversed. Mortality rates were higher for males in all age groups, increasing for males until 85–89 years and then slowing down; for females, it grew at a slower rate throughout (Fig. 1). The age distribution pattern of DALYs was slightly different, both the peaks of males and females appeared in the 70–74 years. For the DALY rate, the male rate decreased after increasing to 85–89 years, while the female rate leveled off after increasing to 75–79 years (Fig. S1).

At the SDI regional level, attributable deaths (141,210) and DALYs (2,286,000) from occupational asbestos exposure were highest in areas with high SDI, as were ASMR and ASDR. Among the 21 GBD regions, Western Europe and high-income North America ranked first and second for deaths or DALYs from occupational asbestos exposure, but the first two ASMR or ASDR occurred in Australasia and Western Europe (Tables 1, S1 and S2).

Among the five diseases, lung cancer, mesothelioma, and ovarian cancer were the three leading causes of death from occupational asbestos exposure, accounting for 97% of the total deaths considered (Table 1). ASMR of the above three diseases also ranked top three (Table 1). A similar pattern was observed by DALYs (Table S1).

**Fig. 2:**
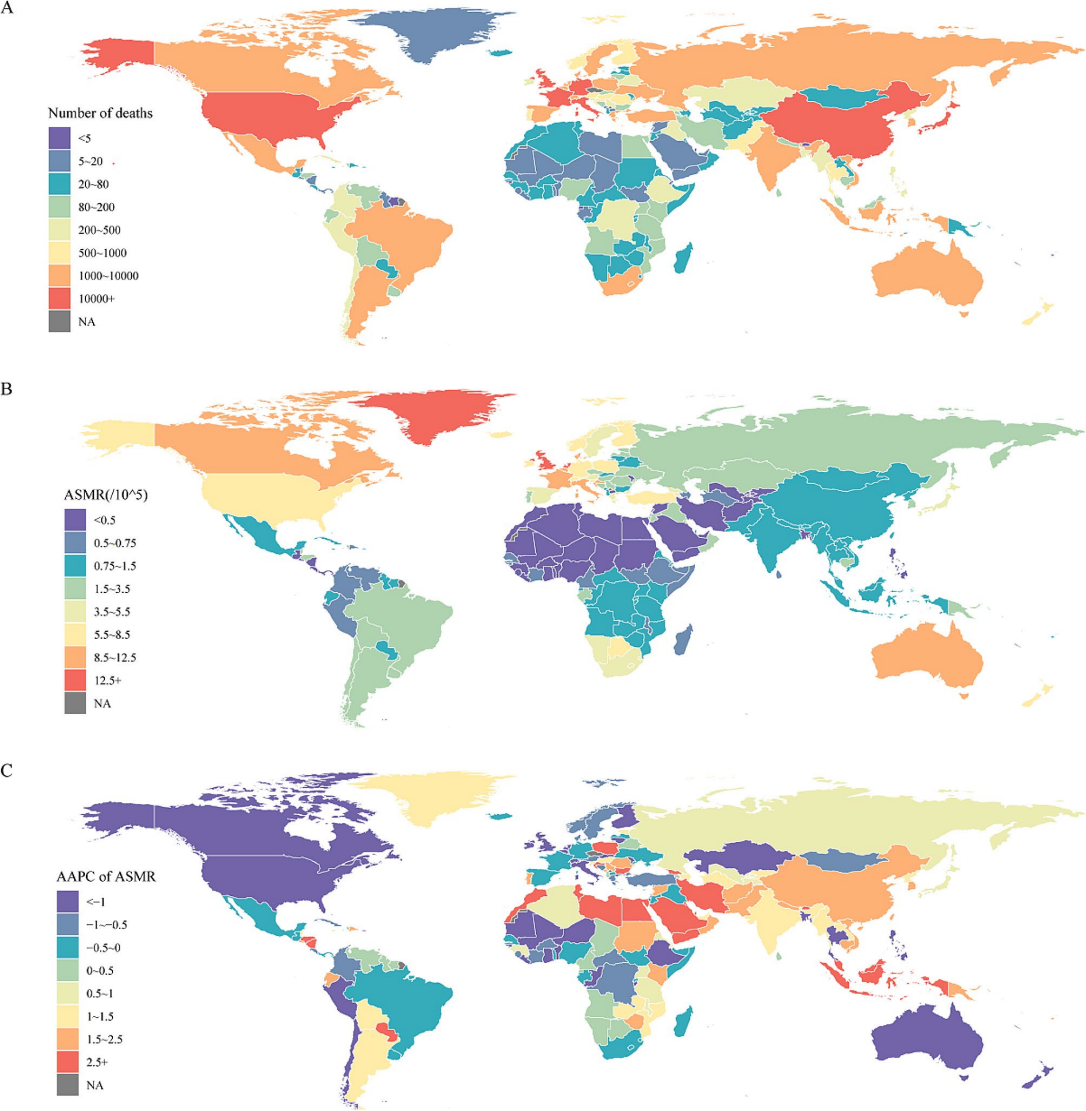
Global deaths attributable to occupational asbestos exposure for both sexes. (**A**) Number of deaths in 2019. (**B**) ASMR in 2019. (**C**) AAPC of ASMR from 1990 to 2019. ASMR, age-standardized mortality rate; AAPC, average annual percentage change; GBD, Global Burden of Disease Study

At the national and regional level, Japan ranked third in the number of deaths caused by occupational asbestos exposure, after the United States and China, and the United Kingdom ranked third in DALYs (Figs. 2A, S2A and Table S3). Greenland and Monaco ranked first and second in terms of ASMR or ASDR (Figs. 2B, S2B and Table S3). Lung cancer was the most common disease in all regions; mesothelioma, ovarian cancer, and asbestosis occurred mainly in eastern sub-Saharan Africa; larynx cancer occurred mainly in South Asia (Fig. S4). DALYs observed a similar distribution pattern (Fig. S5).

### Changing patterns of the global burden of NCDs due to occupational asbestos exposure, 1990–2019

The number of deaths and DALYs due to occupational asbestos exposure increased by 65.65% and 43.66% globally from 1990 to 2019, respectively, with males being the main contributors, accounting for 78.7% and 77.2% of the total increase, respectively (Fig. S6). ASMR and ASDR decreased, with AAPC of − 0.92 (95%CI: − 1.12, − 0.73) and − 1.29 (95%CI: − 1.47, − 1.12), respectively, and the decrease was more significant in males, with AAPC of − 1.13 (95%CI: 1.32, 0.95), − 1.49 (95% CI: − 1.66, − 1.31), respectively (Tables 1, S1 and Fig. S7).

For the SDI region, ASMR or ASDR increased the most in the low-middle SDI region, with AAPC of 1.57(95%CI: 1.23, 1.92) and 1.37(95%CI: 1.04, 1.71), respectively. ASMR or ASDR in high SDI areas decreased the most, and AAPC were − 1.05(95%CI: − 1.2, − 0.89) and − 1.53(95%CI: − 1.71, − 1.36), respectively. For the GBD region, the growth of ASMR or ASDR was the fastest in Central Europe, with AAPC of 2.07 (95%CI: 1.74, 2.41) and 1.8 (95%CI: 1.48, 2.13), respectively. Andean Latin America showed the fastest decline, with AAPC of − 2.42 (95%CI: − 3.16, − 1.68) and − 2.86 (95%CI: − 4.44, − 1.26) (Tables 1 and S1). At the national or regional level, Georgia showed the largest increase in ASMR or ASDR (Figs. 2C, S2C and Table S3).

Among the five diseases, lung cancer, mesothelioma, and ovarian cancer were the top three contributors to the increase in global deaths, accounting for 96.8% of the overall increase. The ASMR of ovarian cancer patients decreased the most, the AAPC was − 1.12 (95%CI: − 1.33, − 0.91), and that of lung cancer and mesothelioma was slightly lower (Table 1).

Similar to the death pattern, the major contribution to the overall increase of DALYs was also the above three diseases, accounting for 75%, 18.5%, and 2.8%, respectively. Lung cancer decreased more rapidly than ovarian cancer (AAPC: − 1.4 (95%CI: − 1.59, − 1.21) vs. − 1.32 (95%CI: − 1.52, − 1.11)), while mesothelioma decreased slightly (AAPC: − 0.60 (95%CI: − 0.73, − 0.46)) (Table S1).

### Global PAF for diseases due to occupational asbestos exposure, 1990–2019

From 1990 to 2019, the global PAF for age-standardized deaths due to occupational asbestos exposure increased from 0.35% (95%UI: 0.27%, 0.45%) to 0.42% (95%UI: 0.31%, 0.52%), AAPC was 0.52 (95%CI: 0.33, 0.72) (Table 1). DALYs-PAF were from 0.15% (95% UI: 0.11%, 0.19%) to 0.16% (95% UI: 0.12%, 0.20%), AAPC was 0.52 (95% CI: 0.03, 0.35) (Table S1).

**Fig. 3:**
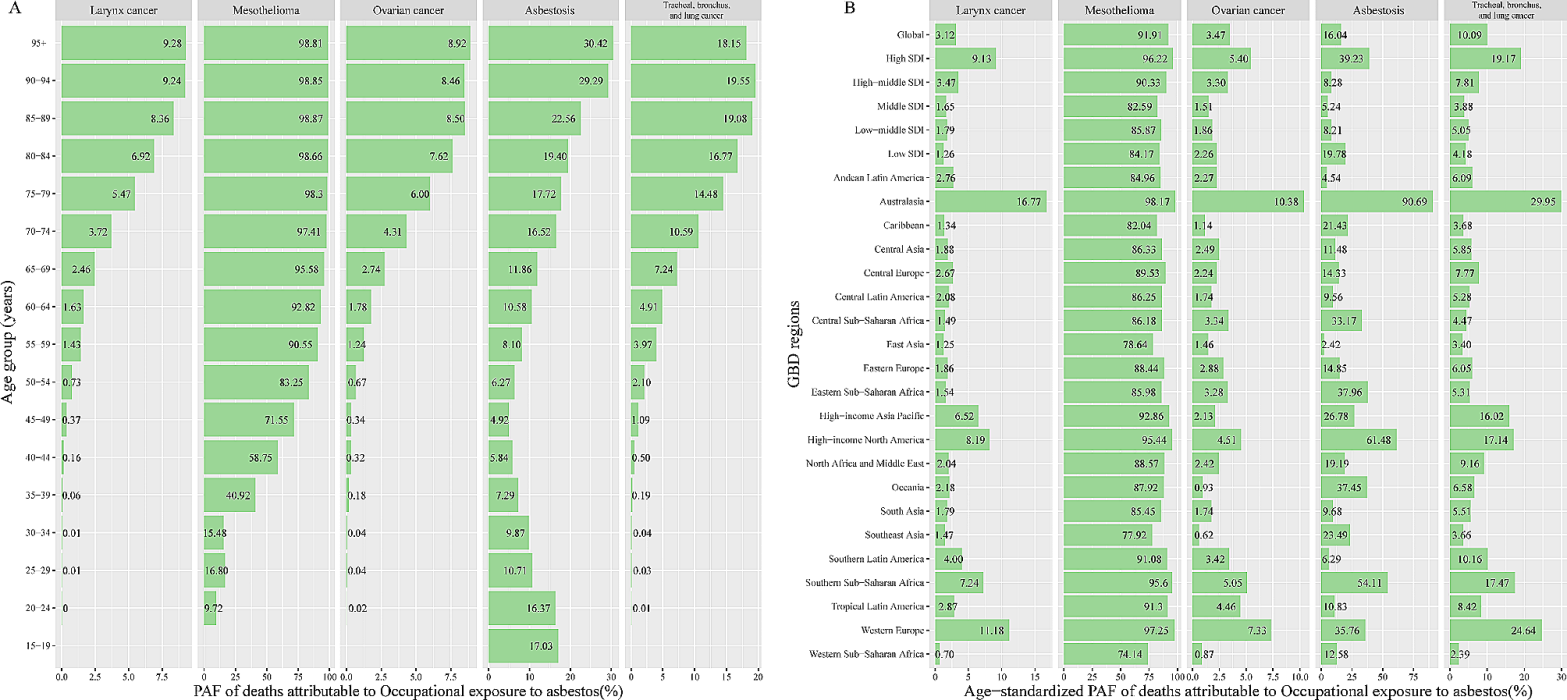
PAF of specific GBD level-three diseases in deaths attributable to occupational asbestos exposure by age and region for both sexes in 2019. (**A**) By age. (**B**) By region. GBD, Global Burden of Disease Study; SDI, socio-demographic index

In five diseases, the age-specific deaths-PAF of larynx cancer and ovarian cancer were positively correlated with age; the PAF for mesothelioma remained stable after increasing to 55–59 years; the PAF of lung cancer showed unimodal age distribution and decreased after increasing to the 90–94 years; the asbestosis distribution was bimodal, with peaks in 15–24 and 90 + years (Fig. 3A). A similar pattern was observed by DALYs-PAF (Fig.S3A).

Mesothelioma, asbestosis, and lung cancer were the top three deaths-PAF. From 1990 to 2019, there was a significant increase in asbestosis (AAPC: 2.39 (95%CI: 2.17, 2.62)), while the deaths-PAF from other diseases showed a downward trend and ovarian cancer showed the fastest decline (AAPC: − 1.04 (95%CI: − 1.2, − 0.88)) (Table 1). A similar pattern was observed by DALYs-PAF (Table S1).

At the level of SDI region, PAF was highest in high SDI regions and lowest in areas with low SDI (Fig. 3B). At the level of GBD, Australasia had the highest PAF and had the highest PAF for all five diseases. West sub-Saharan Africa had the lowest PAF. A similar pattern was observed by DALYs-PAF (Fig. S3B).

### The changing patterns at different SDI levels and baseline burden

**Fig. 4:**
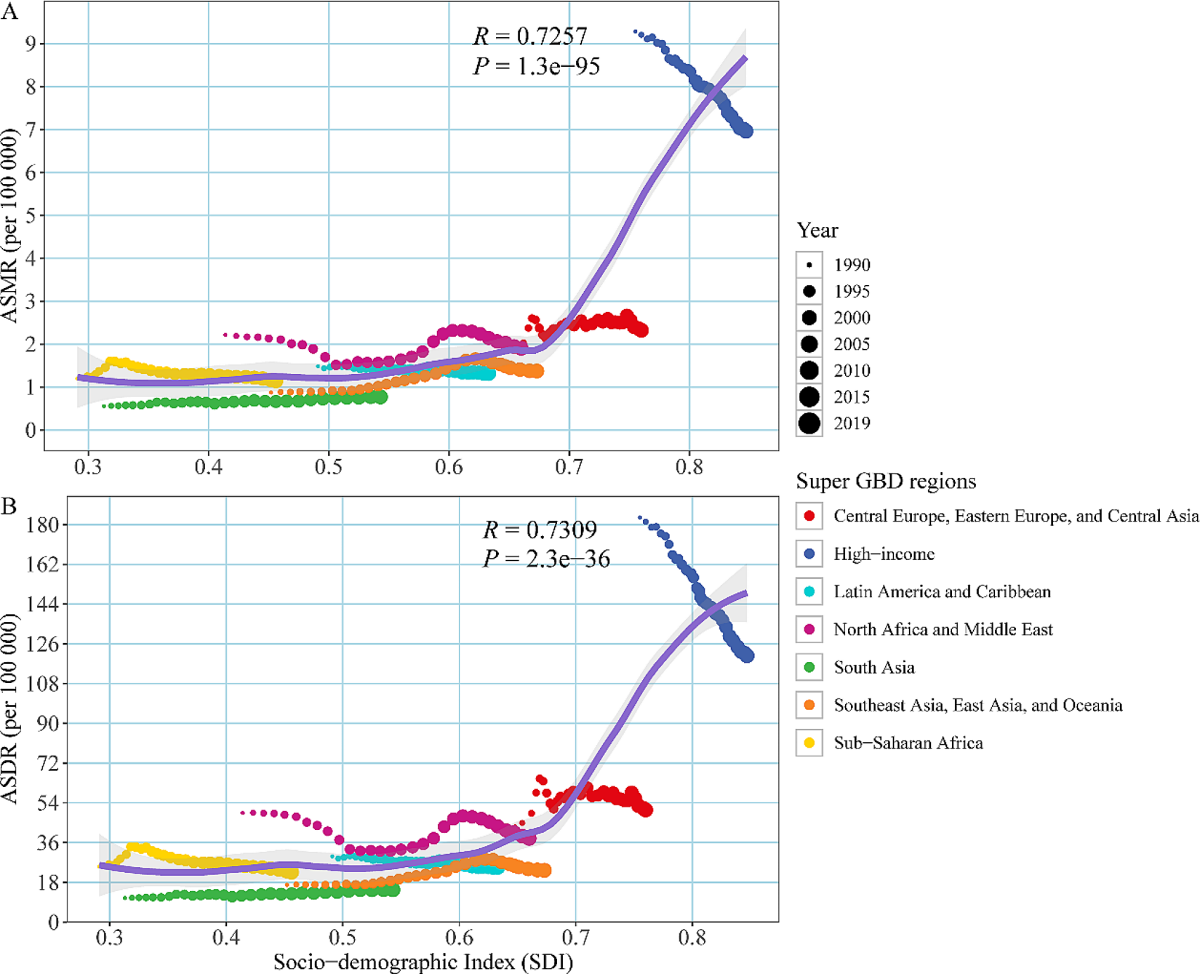
Age-standardized rate attributable to occupational asbestos exposure across 7 Super GBD regions for both sexes, 1990–2019. (**A**) ASMR; (**B**) ASDR. The purple line was an adaptive association fitted with adaptive Loess regression based on all data points. GBD, Global Burden of Disease Study; ASMR, age-standardized mortality rate; ASDR, age-standardized disability-adjusted life year rate

ASMR or ASDR showed an upward trend as SDI increases, with significant increases when SDI > 0.7, with some exceptions. High-income regions experienced a rapid decline in ASMR or ASDR over time, but ASMR/ASDR remained at a high level. The changes in North Africa and the Middle East were non-linear. In South Asia, Southeast Asia, East Asia and Oceania, ASMR or ASDR remained at low levels despite a slow increase (Fig. 4).

**Fig. 5:**
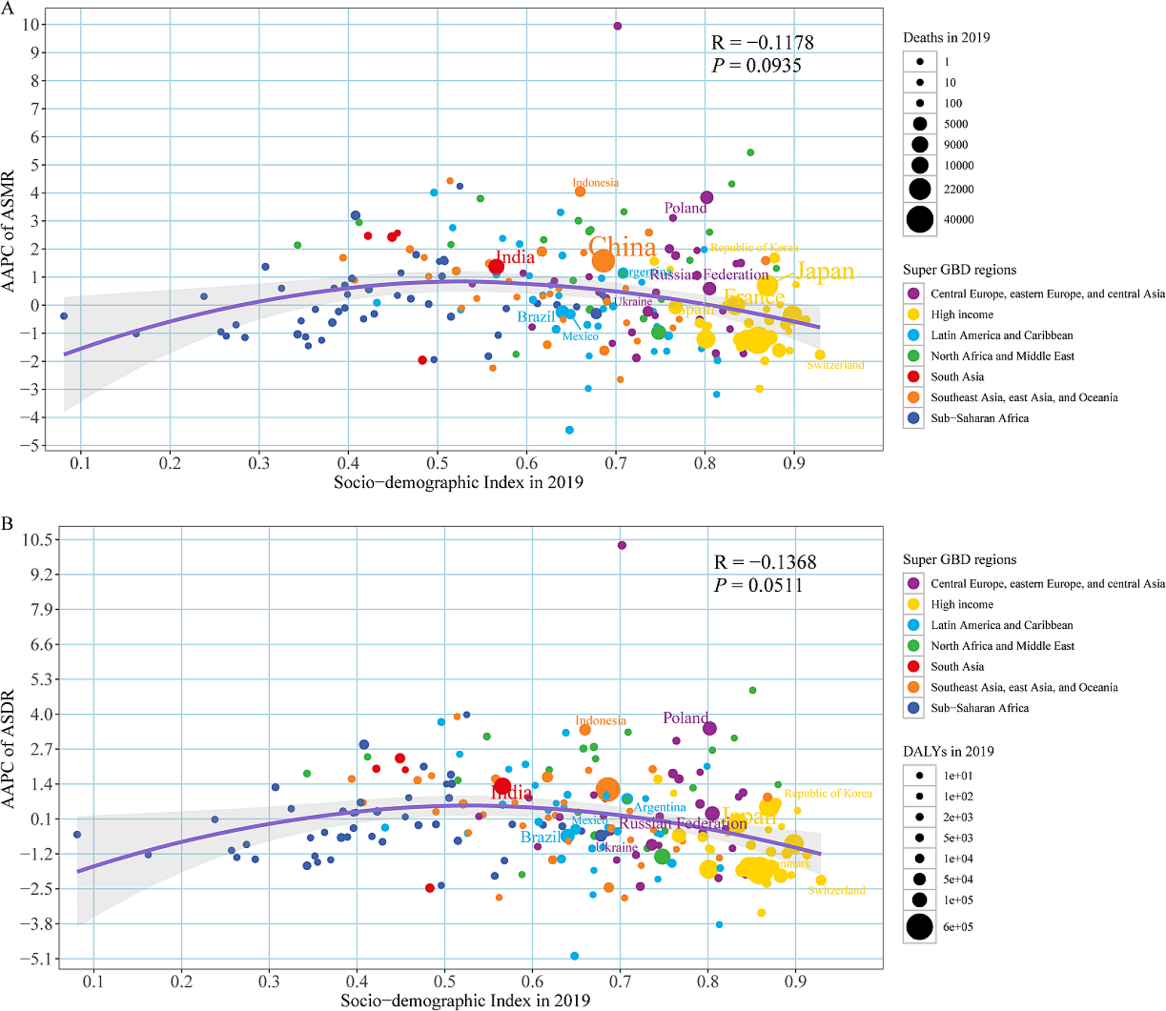
The factors associated with the AAPC of age-standardized rate attributable to occupational asbestos exposure from 1990 to 2019, both sexes, at the national level. (**A**) AAPC of ASMR; (**B**) AAPC of ASDR. The purple line was an adaptive association fitted with adaptive Loess regression based on all data points. ASMR, age-standardized mortality rate; ASDR,age-standardized disability-adjusted life year rate; AAPC, average annual percentage change; SDI, socio-demographic index; GBD, Global Burden of Disease Study

AAPC for ASMR or ASDR was positively correlated with SDI in 2019 (correlation coefficients were 0.09 and 0.05, respectively). In particular, when SDI < 0.6, the rate of change in ASMR or ASDR increased with the increase in SDI, with East Timor in Southeast Asia, East Asia and Oceania growing the fastest. When 0.8 > SDI > 0.6, the growth rate of ASMR or ASDR slowed down. But there were some exceptions, with Bermuda in Latin America and the Caribbean declining the fastest when SDI > 0.8, while Kuwait in North Africa and the Middle East continued to grow rapidly (Fig. 5).

### Global trends and predictions of deaths and ASMR from 1990 to 2035

**Fig. 6:**
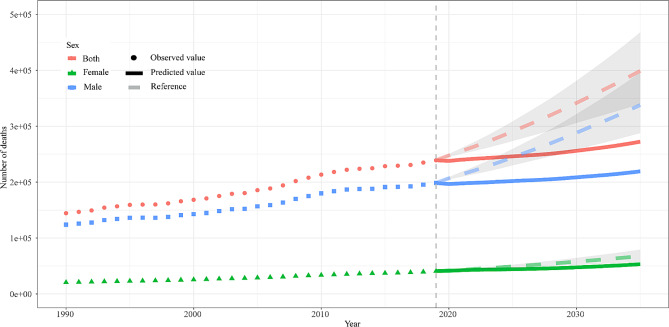
The observed (dashed line) and predicted (solid line) deaths of occupational asbestos exposure from 1990 to 2035. The upper bound of shading represents the rate increased by 1% per year (pessimistic reference) and the lower bound represents decreased by 1% per year (optimistic reference) based on the rate observed in 2019

**Fig. 7:**
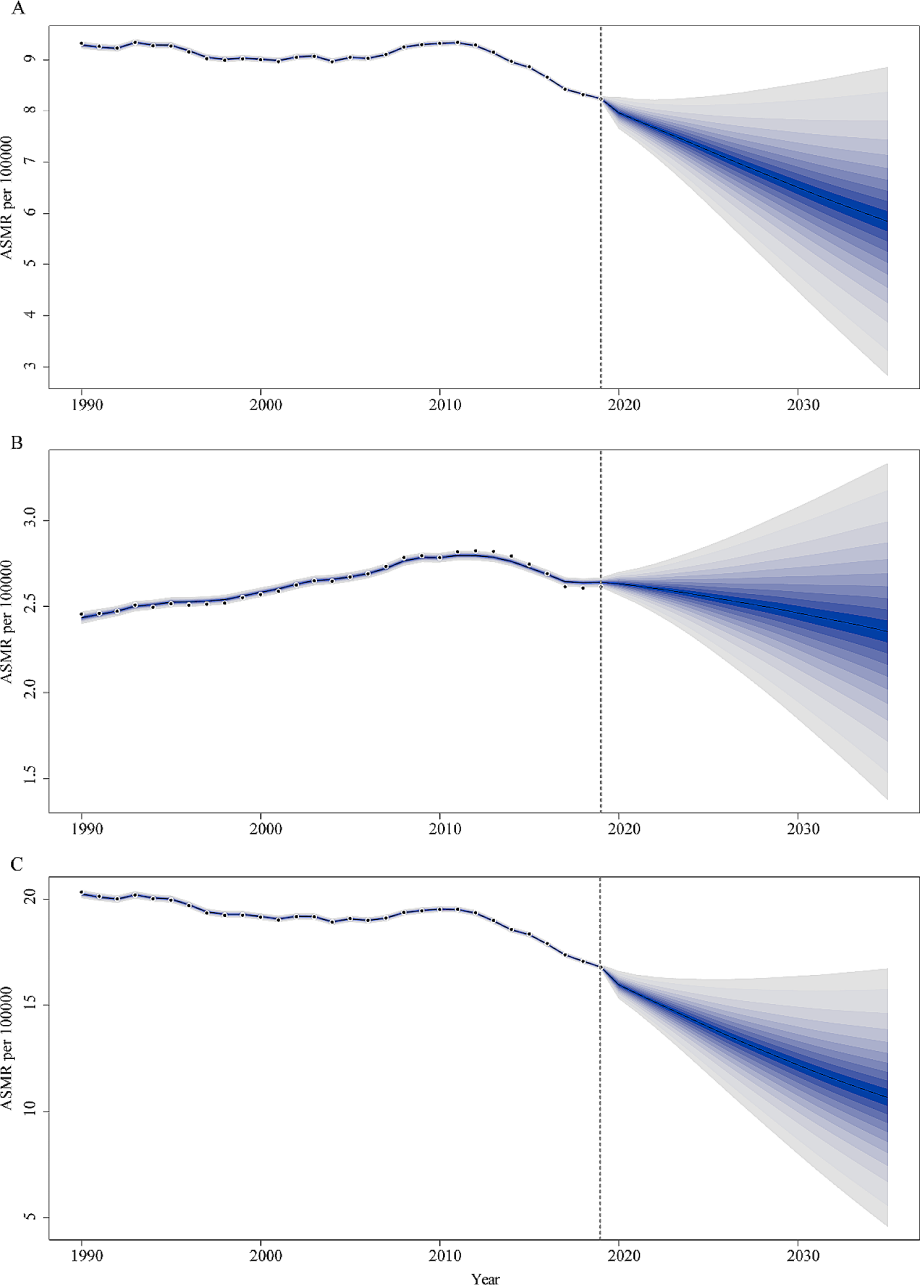
The temporal trends of age-standardized mortality rates (ASMR, per 100,000) of occupational asbestos exposure from 1990 to 2035 at the global level in both sexes (**A**), females (**B**), and males (**C**). The fan shows the predictive distribution between the 5% and 95% quantile, whereby the shaded bands show prediction intervals in increments of 10%. The predictive mean is shown as solid line. Observational values from GBD dataset are shown as filled circles. The vertical dashed line indicates where prediction starts

In this study, the BAPC model was used to predict future mortality trends from occupational asbestos exposure worldwide. The global number of deaths due to occupational asbestos exposure would continue to increase, reaching 2,792,309 by 2035, with 219,235 males and 53,073 females, and males were the main cause of the increase, but the upward trend has slowed (Fig. 6). ASMR was predicted to decline, from 8.24 (95%CI: 8.20, 8.30) to 5.84 (95%CI: 2.82, 8.86) in both sex, from 2.64 (95%CI: 2.62, 2.66) to 2.35 (95%CI: 1.37, 3.33) in females, and from 16.80 (95%CI: 16.70, 16.90) to 10.66 (95%CI: 4.60, 16.72) in males, with a more pronounced decline in males (Fig. 7).

## Discussion

This study provides a comprehensive and systematic description of the disease burden attributed to occupational asbestos exposure. From 1990 to 2019, the burden of NCDs from occupational asbestos exposure has been increasing, with deaths and DALYs nearly tripling, especially among the elderly. Of the five diseases, lung cancer was the leading cause of death, accounting for 83% of the total, while mesothelioma was the most common primary disease in all regions. The NCDs burden was concentrated in high SDI regions such as Western Europe and Australasia. It is important to note that people aged 15–24 years were the primary group for asbestosis, suggesting that the health effects of occupational asbestos exposure may be progressively younger.

The number of deaths and DALYs caused by occupational asbestos exposure worldwide in 2019 mainly occurred in the elderly. We found that DALYs-PAF was the highest in people aged ≥ 85 years, which may be due to the rapid increase in demand for asbestos in the 1940s as industrial expansion followed World War II. At the same time, asbestos, with its unique advantages (simple process manufacturing, low investment cost, superior performance), had become the best choice and was widely used in the construction industry, automobile manufacturing industry, textile industry, shipbuilding industry and other industries, which may lead to people born in this period have a higher probability of exposure to asbestos at a younger age [23, 24]. At the same time, due to the unique pathogenesis of asbestos exposure and the long latency period, people who are exposed at a young age are usually diagnosed as elderly. For example, mesothelioma is a common asbestos-related disease, with over 80% caused by asbestos, its DALYs-PAF ranked first among all diseases in our study. However, the latency period from asbestos exposure to mesothelioma development is as long as 35–40 years [13], and we also found that the DALYs-PAF of mesothelioma in people aged ≥ 85 years maintained a higher level compared to other diseases.

Despite the subsequent decline in asbestos use in industrialized countries and the tightening of occupational environmental exposure limits, this had little effect on workers who accumulated high exposure levels in the early decades of work [25]. It is consistent with the trend in this study of asbestos-related lung, ovarian, and larynx cancer mortality that increases with age starting at age 50. However, we need to consider that the risk of disease after exposure to asbestos does not increase indefinitely, according to the latest research, the incidence of mesothelioma due to asbestos removal in the body will level off after many years of exposure to asbestos [26]. This may also be responsible for the slight decrease in mesothelioma and lung cancer mortality in people aged 90–94 years. It is worth noting that the PAF of asbestosis was higher in adolescents aged 15–24 years, which may be due to the lower diffusion capacity of lung gases in younger, which is more likely to produce asbestosis [27].Increasing genetic susceptibility and/or early exposure to carcinogenic mineral fibers may be responsible for these young patients [28]. It should be taken into account that the burden of NCDs caused by occupational asbestos exposure varies greatly among different populations, and the registration and monitoring tracking system needs to be continuously improved to reduce the health impact of the related disease.

In 2019, the global overall burden of NCDs due to occupational asbestos exposure was higher among males. Compared with females, males are more likely to engage in construction, railway, cement processing, and other industries with high asbestos exposure, with more frequent asbestos exposure [29]. The gender difference in mesothelioma cases is mainly related to occupational exposure and is more susceptible to the effect of asbestos exposure. Unlike occupational exposure in males, non-occupational exposure in females plays a key role in mesothelioma. Influenced by various asbestos exposure factors such as occupation, natural environment and family, females are prone to bias in determining occupational exposure when asbestos-related diseases occur, and social welfare and economic compensation cannot be effectively guaranteed [30]. Therefore, it is necessary to improve the ability to trace the etiology, enhance the awareness of exposure risk, and better protect female health rights and interests. In addition, relevant studies have shown that the mortality rate of asbestosis increased significantly in the environment of occupational asbestos exposure, and the standardized mortality rate was higher in females than that in males [31]. We found that the main contributor to the gender difference was lung cancer, and the association between asbestos and larynx cancer was only observed in males. Males are at a higher risk of exposure to risk factors associated with these cancers [32], such as smoking. At the same time, asbestos fibers can increase the uptake and metabolism of PAHs (one of the most typical carcinogens in cigarette smoke) by lung epithelial cells, while cigarette smoke can increase the binding of asbestos fibers to lung epithelial cells, and the multiplier effect of both may increase the risk of lung cancer [33]. In addition, IARC pointed out that quitting smoking can reduce the incidence of larynx cancer by 90% [34].

Of the disease burden from occupational asbestos exposure, lung cancer, mesothelioma and ovarian cancer are the three leading causes of death. Lung cancer is the first cause of death, and the risk of lung cancer is increased five-fold by exposure to asbestos [35], which may be due to DNA damage caused by asbestos fiber mediated reactive oxygen species and active nitrogen, resulting in tissue inflammation and cell death, and leading to the occurrence of lung cancer [36].Studies have shown that the risk of ovarian cancer is approximately doubled in females with occupational exposure to asbestos, in which inflammatory damage also plays an important role [12]. Mesothelioma tops the PAF list of deaths attributed to occupational asbestos exposure, with 80% of mesothelioma mainly caused by asbestos exposure [13]. In addition, mesothelioma prediction studies have found that due to its long latency period and many factors, its incidence and mortality will continue to increase, and it is expected to peak before 2030, and there will still be a high disease burden in the future [37].

The burden caused by occupational asbestos exposure was positively correlated with SDI as a whole. In areas with SDI greater than 0.8, the burden was higher, but ASMR and ASDR declined faster. In contrast, ASMR and ASDR had risen steadily in low-middle SDI regions (mainly low-middle income countries) [38]. It may be closely related to countries’ level of economic development and the degree to which asbestos bans are enforced. It may also be influenced by other policies: conduct professional training and qualification recognition for workers engaged in the construction and demolition of historic asbestos buildings, accelerate the establishment of occupational asbestos exposure tracking network, conduct health registration and monitoring of past and current asbestos exposed workers and their relatives, research on early biomarkers of asbestos-related diseases such as mesothelioma and lung cancer to achieve early detection and treatment, and promote asbestos substitutes and etc [39]. One study found a co-dependency between the use of asbestos and GDP. The use of asbestos follows the environmental Kuznets curve, the trend of change is first to increase and then to stabilize, and the inflection point appears at 10,000–15,000 GKD (Geary–Khamis Dollars) [40]. High-income countries have reached this tipping point and made the transition away from asbestos, with increased awareness of its carcinogenic risks, bans, and the emergence of asbestos alternatives. However, since that developed countries in the 1970s accumulated a high level of asbestos in the early years to accelerate the transformation and upgrading of industrialization, although the current incidence of the disease has declined in high SDI areas, they still bear most of the global burden of NCDs due to the early accumulation effect. Western Europe has the highest number of deaths and DALYs. Due to the large-scale reconstruction work in Europe after World War II, countries such as Italy and Greece in the region became the main contributors to world asbestos production in the 1980s, accounting for 63% [41]. We found Australasia had the highest ASMR/ASDR and the DALYs- PAF was also much higher than other GBD regions. This may be since that Australia in the region was the world’s largest consumer of asbestos in the 1950s, which was widely used in house building industry. Due to various factors, the implementation of the ban was repeatedly delayed, resulting in an epidemic of asbestos-related diseases in the region that continues to this day [42]. In addition, developing countries, including major asbestos fiber users and producers (such as Russia, Kazakhstan, and India) lack the technology and experience to diagnose mesothelioma, which may have difficulties in diagnosis and is lack of relevant mesothelioma surveillance data, thereby underestimating the risk of the disease. This could lead to a higher disease burden that needs to be dealt with in the coming decades [43]. It also suggests that we should strengthen the sharing of experience and technology in asbestos control between developed and developing countries, and work together to reduce the global burden of asbestos-related diseases.

The global increase in ASMR/ASDR attributed to occupational asbestos exposure from 1990 to 2019 was the fastest in Central Europe, including Georgia. Asbestos has not been completely banned in this area, high per capita asbestos consumption, high exposure level, and potentially high lung cancer incidence have accelerated the rise of its disease burden [44]. Low and middle-income countries are experiencing a development model similar to that of developed countries, and the demand for the use of cheap and durable building materials such as asbestos is becoming more prominent to accelerate infrastructure construction. As the world’s largest consumer of chrysotile asbestos, China, located in the middle SDI region, grew at an annual rate of 7% and suffered a major disease burden in 2019. High exposure levels, inadequate regulatory systems, and slow progress in asbestos replacement may have contributed to the high disease burden.

There are some limitations in this study. The asbestos-related data comes from model reconstructions in the GBD 2019 database and may be biased from the real data. Due to differences in the level of economic development in different parts of the world, the degree of supervision and prevention of asbestos-related diseases is different, so the collection of relevant data is insufficient. Furthermore, the interaction between diseases caused by occupational asbestos exposure and possible potential risks is not sufficiently considered.

## Conclusion

Although ASMR or ASDR from occupational asbestos exposure has declined globally, the burden of NCDs due to a combination of factors such as early accumulation effects, delays in the implementation of the ban and inadequate diagnostic techniques has remained fairly high. As a public health issue of global concern, it is of great significance to call on governments to reduce the use of asbestos, build a more comprehensive regulatory system, and actively seek effective asbestos substitutes to reduce the risk of disease. At the same time, it is necessary to fully consider the susceptibility of the elderly, the lag of asbestos onset, the uniqueness of the working environment, and the urgency of the development of low-income countries, and to formulate more appropriate joint prevention and control strategies as soon as possible.

### Electronic supplementary material

Below is the link to the electronic supplementary material.

Supplementary Material 1

Supplementary Material 2

Supplementary Material 3

Supplementary Material 4

Supplementary Material 5

Supplementary Material 6

Supplementary Material 7

Supplementary Material 8

Supplementary Material 9

Supplementary Material 10

## Data Availability

All data generated or analyzed during this study are included in this published article and its supplementary information files. All data can be extracted from the online GBD repository, https://ghdx.healthdata.org/gbd-2019.
