# Supplementary material for: Global, regional, and national burden of non-communicable diseases attributable to occupational asbestos exposure 1990–2019 and prediction to 2035: worsening or improving?
Source: BMC Public Health. 2024 Mar 18;24:832. doi: 10.1186/s12889-024-18099-4 (PMC10946175; doi:10.1186/s12889-024-18099-4)
Supplement: Supplementary file 1 — Supplementary Material 1 [file 12889_2024_18099_MOESM1_ESM.docx]

Table S1. Global DALYs attributable to occupational exposure to asbestos in 1990 and 2019, and the temporal trend from 1990 to 2019.

| **Cause of DALYs** | **1990** | | |  | **2019** | | |  | **1990-2019** | |
| --- | --- | --- | --- | --- | --- | --- | --- | --- | --- | --- |
|  | **DALYs**  **No.×105**  **（95%UI）** | **ASDR per**  **100 000**  **(95%UI)** | **Age-standardized**  **PAF, %**  **(95%UI)** |  | **DALYs**  **No.×105**  **（95%UI）** | **ASDR per**  **100 000**  **(95%UI)** | **Age-standardized**  **PAF, %**  **(95%UI)** |  | **AAPC of**  **ASDR**  **(95%CI)** | **AAPC of Age-**  **standardized**  **PAF(95%CI)** |
| **Overall** | 29.16 (21.63 ,36.93) | 75.32 (55.94 ,95.25) | 0.15 (0.11 , 0.19) |  | 41.89 (31.27 , 53.2) | 51.77 (38.71 ,65.65) | 0.16 (0.12 , 0.2) |  | -1.29 (-1.47 , -1.12) | 0.16 (-0.03 , 0.35) |
| **Sex** |  |  |  |  |  |  |  |  |  |  |
| Female | 4.14  (2.85 , 5.52) | 19.41 (13.48 ,25.78) | 0.04 (0.03 , 0.06) |  | 7.04 (4.67 , 9.05) | 16.08 (10.67 ,20.68) | 0.05 (0.04 , 0.07) |  | -0.64 (-0.81 , -0.48) | 0.8 (0.59 , 1.02) |
| Male | 25.02 (17.76 ,32.66) | 147.2 (105.28 ,190.56) | 0.27 (0.19 , 0.36) |  | 34.85 (24.54 , 45.63) | 95.45 (67.75 ,124.58) | 0.27 (0.19 , 0.35) |  | -1.49 (-1.66 , -1.31) | -0.04 (-0.24 , 0.15) |
| **Disease type** |  |  |  |  |  |  |  |  |  |  |
| **All** | 29.16 (21.63,36.93) | 75.32 (55.94,95.25) | 0.3 (0.22 , 0.38) |  | 41.89 (31.27,53.2) | 51.77 (38.71,65.65) | 0.26 (0.19 , 0.33) |  | -1.29 (-1.47 , -1.12) | -0.56 (-0.71 , -0.4) |
| **Neoplasms** | 28.78 (21.25,36.51) | 74.37 (55.01,94.17) | 1.95 (1.43 , 2.47) |  | 41.18 (30.59,52.43) | 50.88 (37.85,64.68) | 1.66 (1.23 , 2.12) |  | -1.31 (-1.48 , -1.14) | -0.54 (-0.77 , -0.32) |
| Larynx cancer | 0.58 (0.32,0.88) | 1.49 (0.82,2.24) | 2.53 (1.39 , 3.8) |  | 0.7 (0.38,1.06) | 0.86 (0.47,1.3) | 2.21 (1.23 , 3.37) |  | -1.86 (-2.03 , -1.68) | -0.45 (-0.63 , -0.27) |
| Tracheal, bronchus, and lung cancer | 24.1 (16.82,31.69) | 62.6 (43.98,81.92) | 9.52 (6.6 , 12.42) |  | 33.65 (23.36,44.51) | 41.71 (29.02,54.99) | 7.57 (5.24 , 9.96) |  | -1.4 (-1.59 , -1.21) | -0.79 (-0.88 , -0.7) |
| Ovarian cancer | 0.77 (0.35,1.26) | 2.04 (0.93,3.31) | 3.16 (1.43 , 5.09) |  | 1.13 (0.5,1.85) | 1.4 (0.62,2.28) | 2.18 (0.99 , 3.52) |  | -1.32 (-1.52 , -1.11) | -1.29 (-1.45 , -1.12) |
| Mesothelioma | 3.33 (2.86,3.83) | 8.24 (7.19,9.34) | 87.31 (84.66 , 89.84) |  | 5.69 (5.1,6.17) | 6.91 (6.18,7.48) | 85.2 (82.17 , 87.99) |  | -0.6 (-0.73 , -0.46) | -0.08 (-0.1 , -0.06) |
| **Chronic respiratory diseases** | 0.38 (0.3,0.46) | 0.95 (0.76,1.14) | 0.05 (0.04 , 0.05) |  | 0.71 (0.54,0.82) | 0.89 (0.68,1.02) | 0.07 (0.05 , 0.08) |  | -0.18 (-0.33 , -0.04) | 1.5 (1.37 , 1.63) |
| Asbestosis | 0.38 (0.3,0.46) | 0.95 (0.76,1.14) | 4.77 (3.83 , 5.8) |  | 0.71 (0.54,0.82) | 0.89 (0.68,1.02) | 8.06 (6.1 , 9.61) |  | -0.18 (-0.33 , -0.04) | 1.87 (1.68 , 2.07) |
| **Location** |  |  |  |  |  |  |  |  |  |  |
| **SDI region** |  |  |  |  |  |  |  |  |  |  |
| High SDI | 19.25 (14.3,24.06) | 179.94 (133.69,225.39) | 0.66 (0.49 , 0.84) |  | 22.86 (17.27,28.31) | 115.91 (87.46,143.6) | 0.55 (0.41 , 0.7) |  | -1.53 (-1.71 , -1.36) | -0.67 (-0.8 , -0.53) |
| High-middle SDI | 7.07 (5.16,9.13) | 64.99 (47.47,83.82) | 0.18 (0.13 , 0.23) |  | 10.61 (7.6,13.97) | 51.39 (36.85,67.48) | 0.21 (0.15 , 0.28) |  | -0.79 (-1.2 , -0.38) | 0.66 (0.41 , 0.91) |
| Middle SDI | 1.79 (1.29,2.42) | 17.82 (12.77,24.22) | 0.04 (0.03 , 0.05) |  | 5.29 (3.75,7.23) | 21.95 (15.57,29.98) | 0.08 (0.05 , 0.1) |  | 0.72 (0.56 , 0.87) | 2.27 (2.12 , 2.41) |
| Low-middle SDI | 0.73 (0.48,1.13) | 12.45 (8.4,19.39) | 0.02 (0.01 , 0.03) |  | 2.45 (1.76,3.24) | 18.37 (13.21,24.26) | 0.05 (0.03 , 0.06) |  | 1.37 (1.04 , 1.71) | 3.15 (2.91 , 3.4) |
| Low SDI | 0.3 (0.15,0.71) | 12.54 (6.37,30.38) | 0.01 (0.01 , 0.04) |  | 0.66 (0.39,1.41) | 13.35 (7.94,28.51) | 0.03 (0.02 , 0.06) |  | 0.21 (0.09 , 0.33) | 2.17 (2.03 , 2.3) |
| **GBD Region** |  |  |  |  |  |  |  |  |  |  |
| Andean Latin America | 0.08 (0.04,0.11) | 38.66 (20.07,57.74) | 0.09 (0.05 , 0.13) |  | 0.09 (0.05,0.14) | 16.28 (9.92,25.06) | 0.06 (0.04 , 0.09) |  | -2.86 (-4.44 , -1.26) | -1.04 (-1.88 , -0.19) |
| Australasia | 0.68 (0.54,0.83) | 283.25 (221.05,341.74) | 1.07 (0.82 , 1.33) |  | 0.85 (0.68,1.01) | 165.49 (132.33,197.88) | 0.84 (0.64 , 1.06) |  | -1.81 (-2.06 , -1.56) | -0.84 (-1.04 , -0.63) |
| Caribbean | 0.06 (0.04,0.08) | 22.76 (15.73,31.59) | 0.05 (0.04 , 0.07) |  | 0.1 (0.07,0.15) | 19.58 (12.82,28.82) | 0.06 (0.04 , 0.08) |  | -0.41 (-1.24 , 0.43) | -0.12 (-0.62 , 0.38) |
| Central Asia | 0.18 (0.11,0.27) | 35.63 (22.71,54.22) | 0.08 (0.05 , 0.13) |  | 0.22 (0.15,0.31) | 30.43 (20.7,41.59) | 0.09 (0.06 , 0.12) |  | -0.51 (-1.1 , 0.09) | 0.19 (-0.43 , 0.82) |
| Central Europe | 0.67 (0.45,0.93) | 43.16 (29.18,59.81) | 0.12 (0.08 , 0.17) |  | 1.6 (1.04,2.31) | 72.77 (47.2,105.12) | 0.3 (0.2 , 0.43) |  | 1.8 (1.48 , 2.13) | 3.17 (2.8 , 3.55) |
| Central Latin America | 0.15 (0.11,0.19) | 18.54 (13.89,23.9) | 0.05 (0.04 , 0.06) |  | 0.41 (0.29,0.55) | 17.43 (12.56,23.49) | 0.06 (0.05 , 0.08) |  | -0.26 (-1.16 , 0.65) | 0.81 (0.01 , 1.61) |
| Central Sub-Saharan Africa | 0.06 (0.02,0.18) | 24.5 (9.62,80.84) | 0.03 (0.01 , 0.09) |  | 0.11 (0.04,0.32) | 20.51 (7.85,62.44) | 0.04 (0.02 , 0.12) |  | -0.61 (-0.8 , -0.42) | 1.2 (1.06 , 1.35) |
| East Asia | 1.42 (0.92,2.14) | 17.04 (11.39,25.39) | 0.04 (0.03 , 0.06) |  | 4.97 (3.29,7.23) | 24.55 (16.38,35.4) | 0.11 (0.07 , 0.16) |  | 1.2 (0.95 , 1.44) | 3.38 (3.13 , 3.62) |
| Eastern Europe | 1.3 (0.88,1.79) | 44.45 (30.05,62.04) | 0.12 (0.08 , 0.17) |  | 1.44 (0.96,2) | 41.52 (27.8,57.86) | 0.13 (0.09 , 0.18) |  | -0.11 (-1.63 , 1.43) | 0.53 (-0.55 , 1.64) |
| Eastern Sub-Saharan Africa | 0.13 (0.05,0.39) | 17.26 (7.17,53.04) | 0.02 (0.01 , 0.06) |  | 0.26 (0.11,0.8) | 16.46 (7.15,51.84) | 0.03 (0.01 , 0.11) |  | -0.18 (-0.28 , -0.07) | 2.17 (1.89 , 2.45) |
| High-income Asia Pacific | 1.14 (0.8,1.5) | 57.43 (40.52,75.63) | 0.25 (0.17 , 0.33) |  | 3.16 (2.2,4.19) | 62.73 (44.03,83.16) | 0.39 (0.27 , 0.52) |  | 0.27 (0.01 , 0.54) | 1.57 (1.33 , 1.81) |
| High-income North America | 7.15 (5.19,9.08) | 196.38 (142.14,250.86) | 0.68 (0.49 , 0.88) |  | 7.54 (5.6,9.61) | 114.8 (85.36,146.21) | 0.45 (0.32 , 0.59) |  | -1.8 (-1.99 , -1.62) | -1.37 (-1.55 , -1.19) |
| North Africa and Middle East | 0.87 (0.54,1.3) | 49.4 (31.37,72.26) | 0.1 (0.06 , 0.15) |  | 1.57 (0.95,2.46) | 37.93 (23.07,58.42) | 0.12 (0.07 , 0.18) |  | -0.96 (-1.3 , -0.61) | 0.52 (0.1 , 0.94) |
| Oceania | 0.01 (0.01,0.01) | 27.26 (17.02,44.06) | 0.05 (0.03 , 0.08) |  | 0.03 (0.01,0.04) | 38.47 (21.97,64.12) | 0.08 (0.05 , 0.12) |  | 1.19 (1.03 , 1.35) | 1.56 (1.29 , 1.83) |
| South Asia | 0.58 (0.37,0.97) | 10.91 (7.03,17.78) | 0.02 (0.01 , 0.03) |  | 2 (1.4,2.79) | 14.67 (10.35,20.33) | 0.04 (0.03 , 0.05) |  | 1.13 (0.5 , 1.77) | 3.05 (2.4 , 3.69) |
| Southeast Asia | 0.43 (0.29,0.61) | 15.93 (10.49,22.78) | 0.03 (0.02 , 0.05) |  | 1.12 (0.72,1.66) | 18.69 (12.01,27.61) | 0.06 (0.04 , 0.08) |  | 0.59 (0.35 , 0.84) | 2.17 (1.93 , 2.41) |
| Southern Latin America | 0.25 (0.17,0.34) | 52.77 (35.9,71.78) | 0.16 (0.11 , 0.23) |  | 0.47 (0.31,0.65) | 55.4 (37.18,76.71) | 0.23 (0.15 , 0.32) |  | 0.24 (-0.23 , 0.71) | 1.26 (0.95 , 1.58) |
| Southern Sub-Saharan Africa | 0.27 (0.19,0.37) | 98.58 (68.82,137.68) | 0.18 (0.13 , 0.25) |  | 0.49 (0.37,0.63) | 90.36 (68.09,115.12) | 0.17 (0.13 , 0.22) |  | -0.27 (-1.14 , 0.6) | -0.21 (-0.92 , 0.51) |
| Tropical Latin America | 0.37 (0.28,0.48) | 40.54 (31.01,51.37) | 0.09 (0.07 , 0.11) |  | 0.85 (0.67,1.06) | 35.36 (27.69,44.02) | 0.12 (0.09 , 0.15) |  | -0.48 (-0.94 , -0.02) | 1.05 (0.68 , 1.43) |
| Western Europe | 13.3 (10.02,16.44) | 226.77 (170.41,281.15) | 0.87 (0.65 , 1.11) |  | 14.5 (11.13,17.91) | 158.86 (121,197.37) | 0.83 (0.61 , 1.07) |  | -1.24 (-1.35 , -1.13) | -0.18 (-0.34 , -0.02) |
| Western Sub-Saharan Africa | 0.07 (0.04,0.14) | 8.39 (4.59,15.65) | 0.01 (0.01 , 0.02) |  | 0.13 (0.07,0.23) | 6.94 (4.06,12.91) | 0.01 (0.01 , 0.02) |  | -0.63 (-0.8 , -0.46) | 0.86 (0.61 , 1.1) |

1. ASDR, age-standardized DALY rate;
2. PAF, population attributable fraction;
3. AAPC, average annual percentage change;
4. UI, uncertainty interval;
5. CI, confidence interval
