# Supplementary figures and images for: Global, regional, and national burden of non-communicable diseases attributable to occupational asbestos exposure 1990–2019 and prediction to 2035: worsening or improving?
Source: BMC Public Health. 2024 Mar 18;24:832. doi: 10.1186/s12889-024-18099-4 (PMC10946175; doi:10.1186/s12889-024-18099-4)

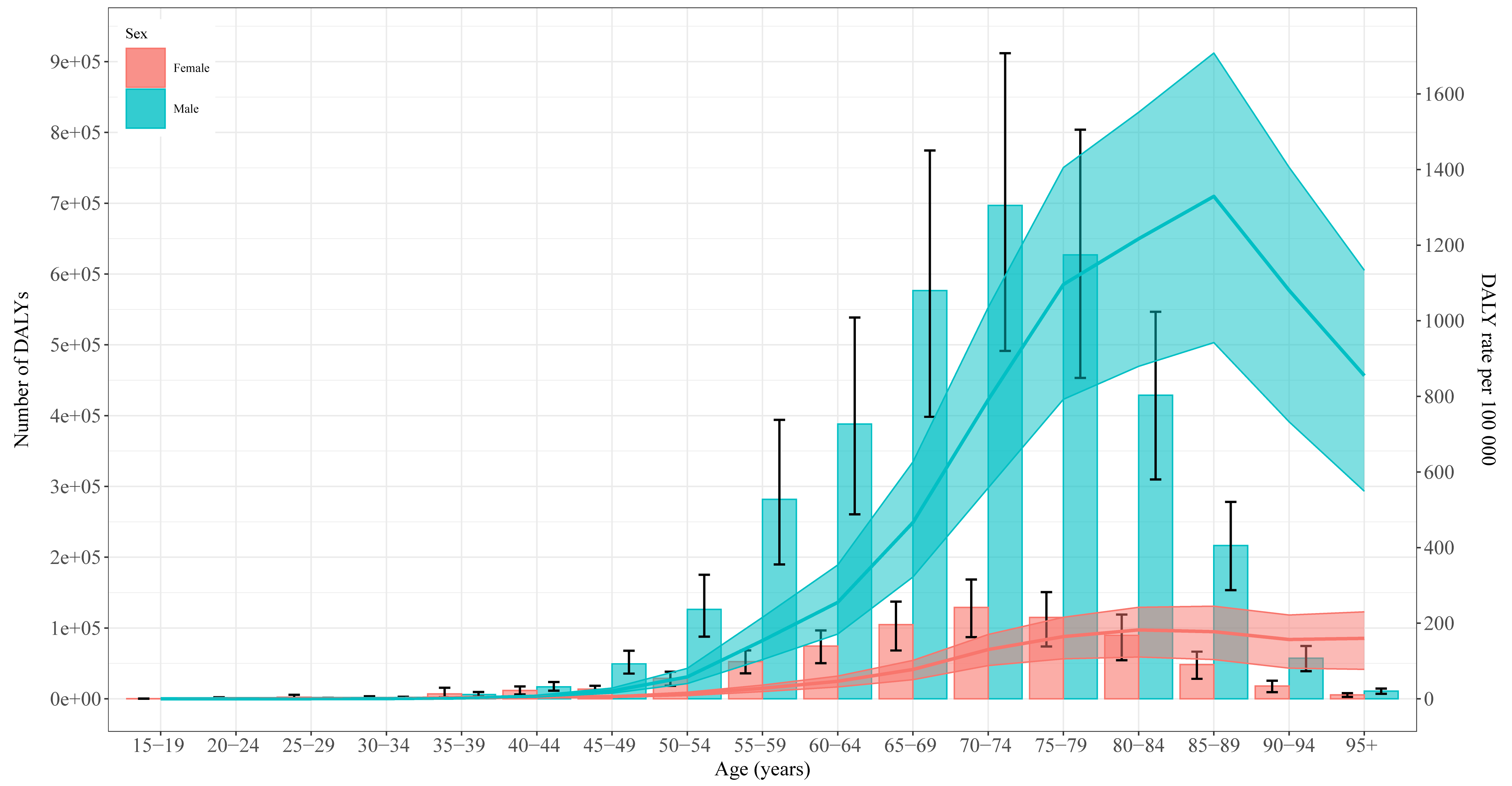

Supplement: Supplementary file 4 — Supplementary Material 4 [file 12889_2024_18099_MOESM4_ESM.png]

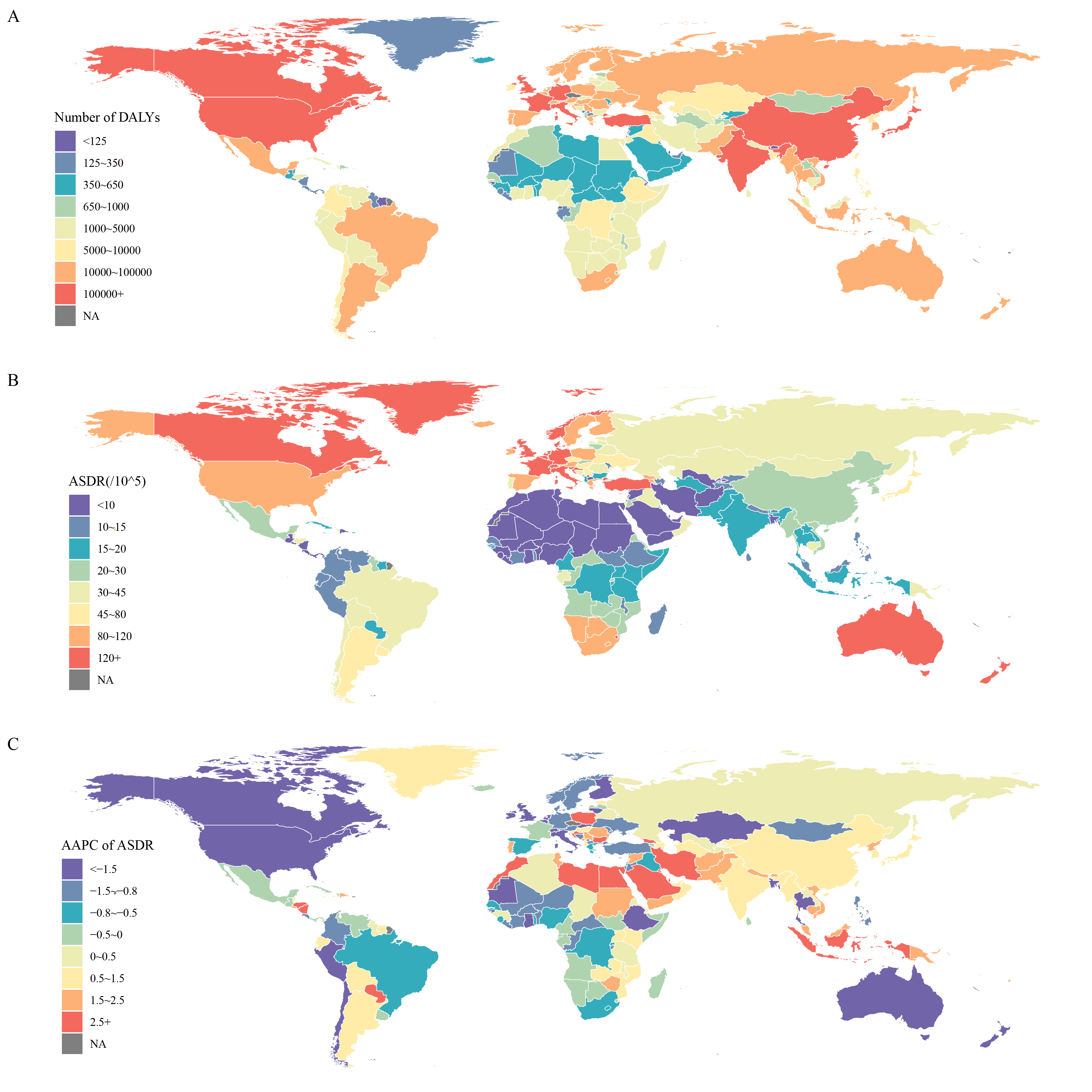

Supplement: Supplementary file 5 — Supplementary Material 5 [file 12889_2024_18099_MOESM5_ESM.png]

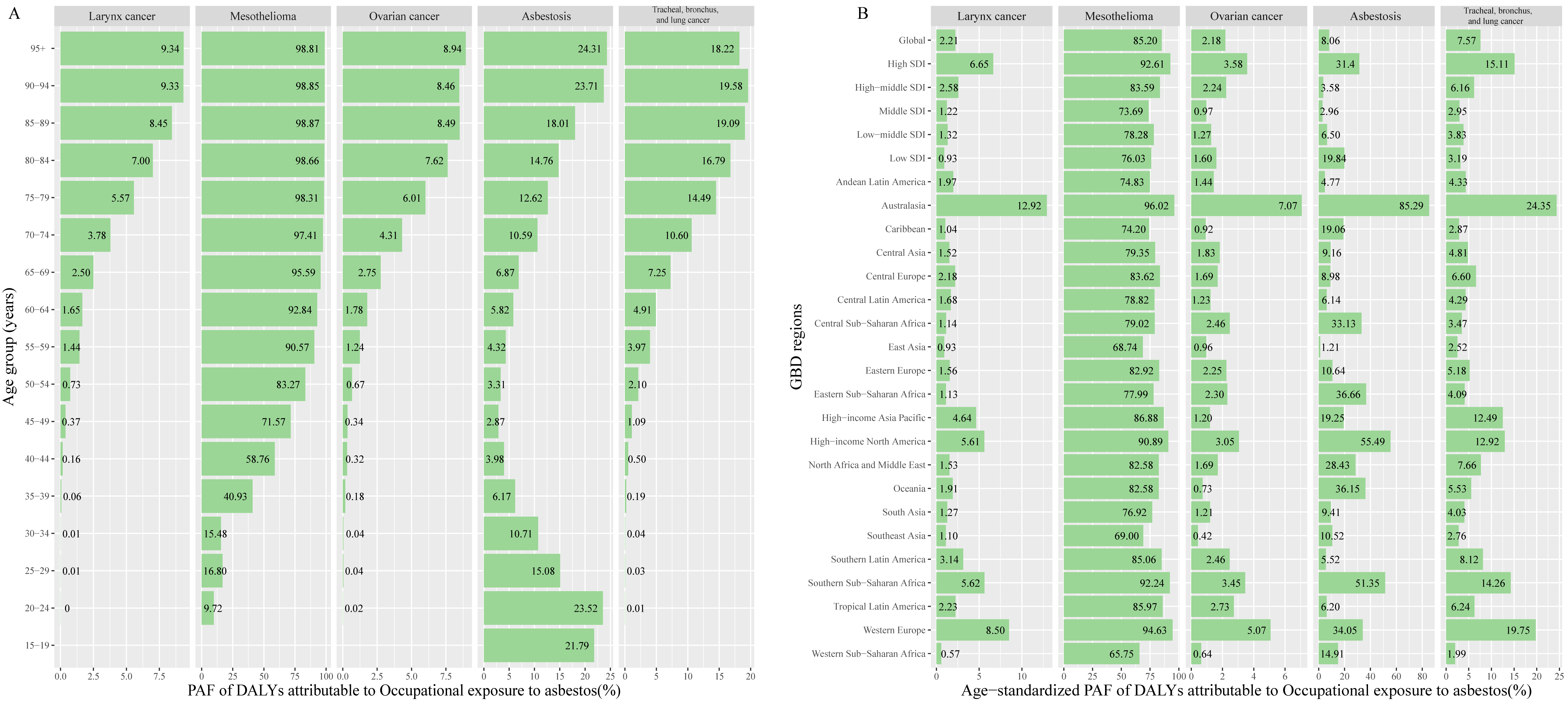

Supplement: Supplementary file 6 — Supplementary Material 6 [file 12889_2024_18099_MOESM6_ESM.png]

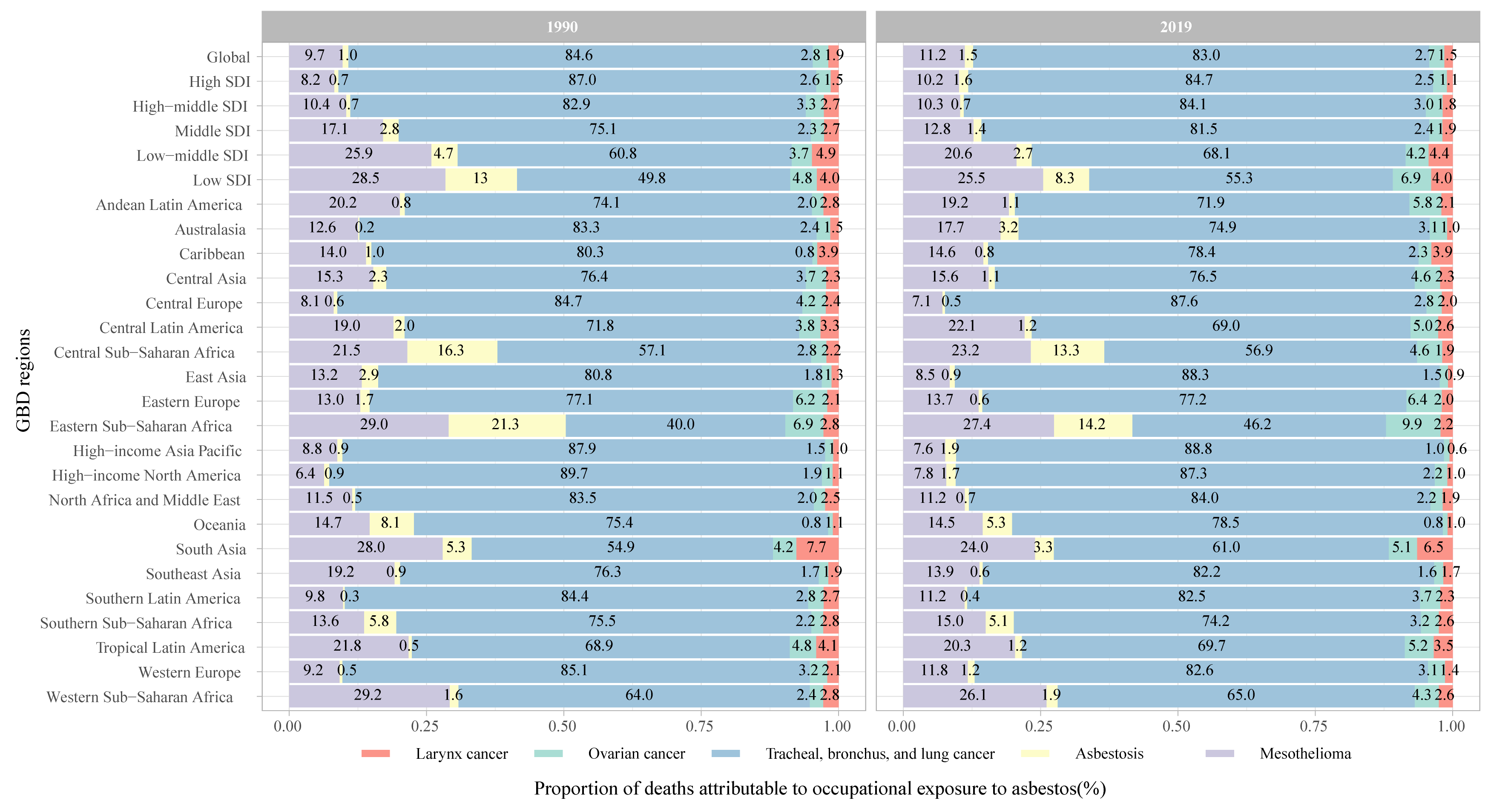

Supplement: Supplementary file 7 — Supplementary Material 7 [file 12889_2024_18099_MOESM7_ESM.png]

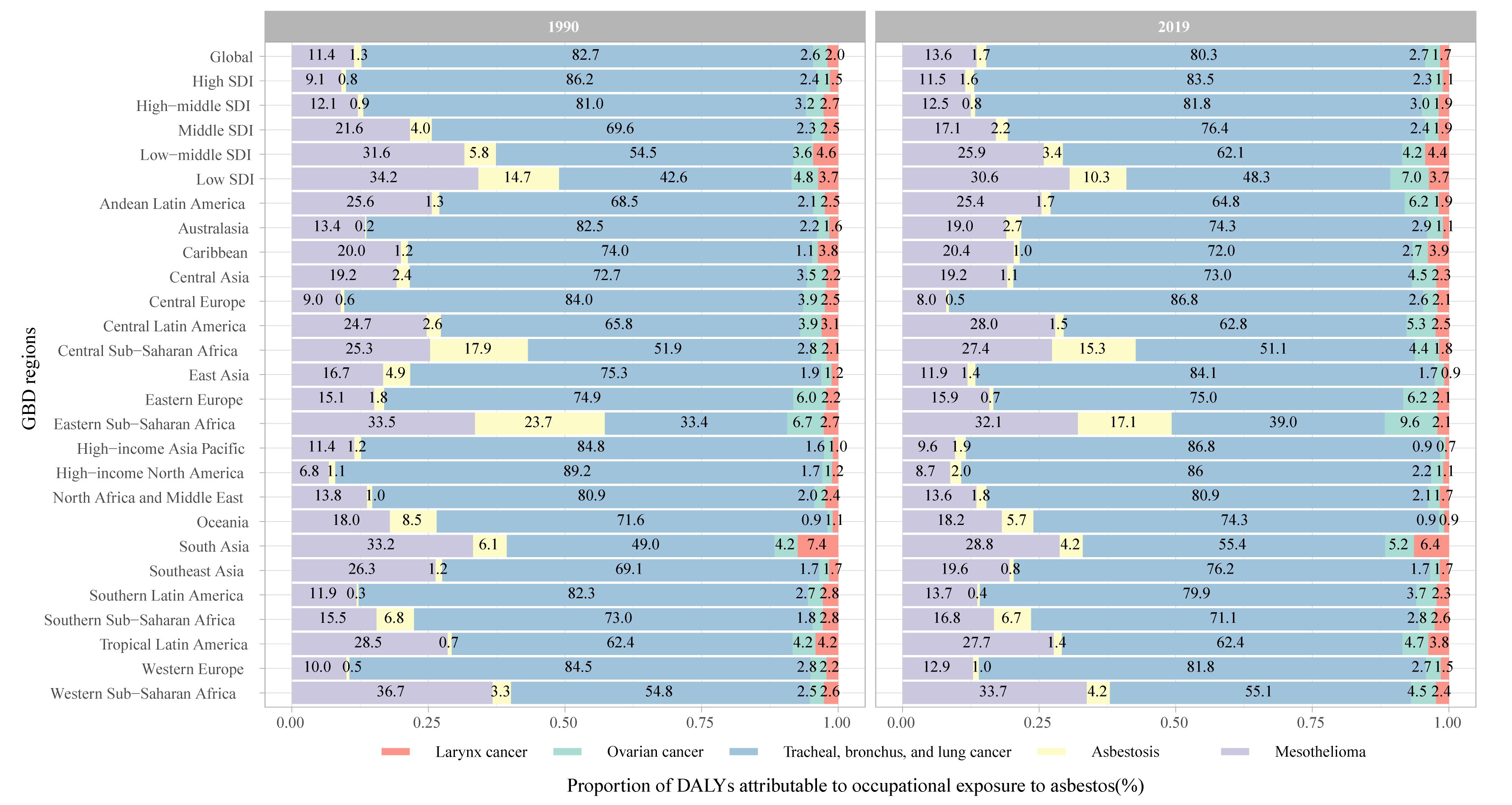

Supplement: Supplementary file 8 — Supplementary Material 8 [file 12889_2024_18099_MOESM8_ESM.png]

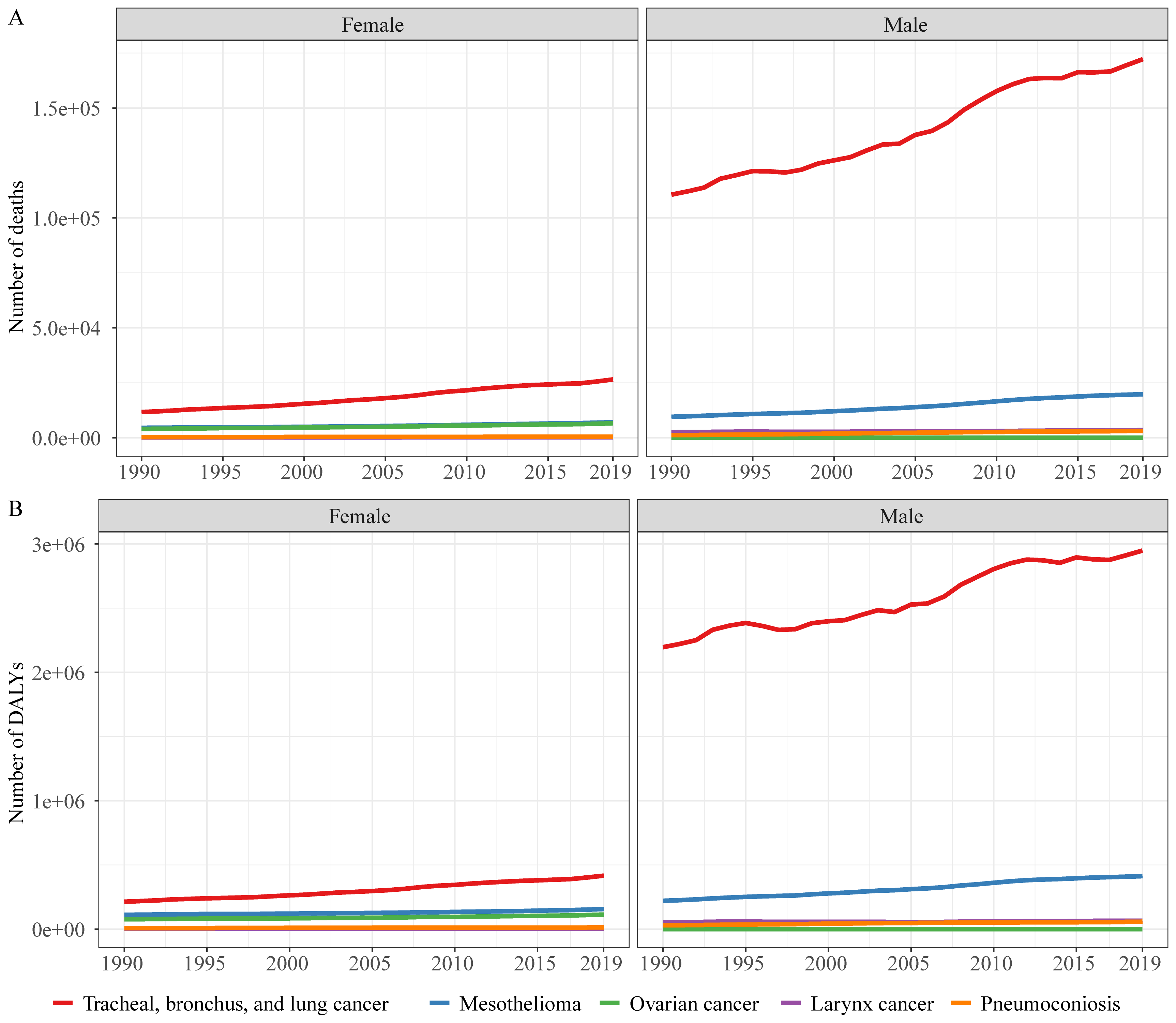

Supplement: Supplementary file 9 — Supplementary Material 9 [file 12889_2024_18099_MOESM9_ESM.png]

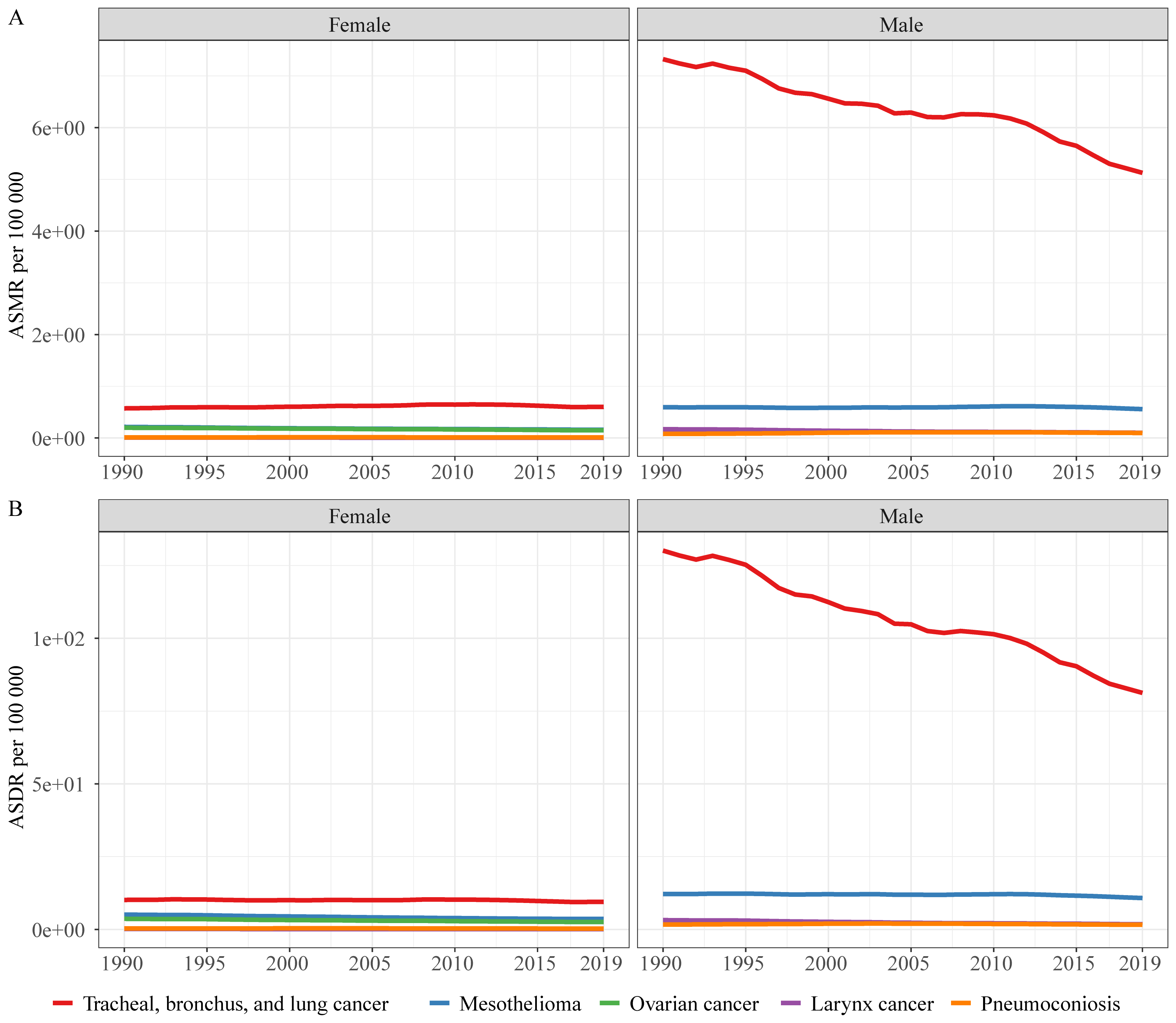

Supplement: Supplementary file 10 — Supplementary Material 10 [file 12889_2024_18099_MOESM10_ESM.png]
